# Supplementary material for: Sox9 and Sox8 protect the adult testis from male-to-female genetic reprogramming and complete degeneration
Source: eLife. 2016 Jun 21;5:e15635. doi: 10.7554/eLife.15635 (PMC4945155; doi:10.7554/eLife.15635)
Supplement: Supplementary file 1. — DOI: http://dx.doi.org/10.7554/eLife.15635.031 [file elife-15635-supp1.doc]

**Complete lists of genes included in the 8 molecular pathways mentioned in Figure 2-figure supplement 2.**

**Sertoli-Sertoli Cell Junction Dynamics**

*A2m, Acta1, Acta2, Actb, Actg1, Actg2, Actn1, Actn2, Actn3, Actn4, Actrt1, Adcy10, Akt1, Akt2, Akt3, Araf, Arl6ip1, Axin1, Bcar1, Bp2, Braf, Cdc42, Cdh1, Cfl1, Cfl2, Cgn, Cldn1, Cldn10, Cldn11, Cldn12, Cldn14, Cldn15, Cldn16, Cldn17, Cldn18, Cldn19, Cldn2, Cldn20, Cldn23, Cldn3, Cldn4, Cldn5, Cldn6, Cldn7, Cldn8, Cldn9, Ctnna1, Ctnna2, Ctnna3, Ctnnb1, Ctnnd1, Dab2ip, Ensg00000183311, Ensg00000224156, Ensg00000227739, Ensg00000229684,Ensg00000232421, Ensg00000232575, Ensg00000235067, Epb41, Epn1, Epn2, Epn3, F11r, Feom3, Gsk3a, Gsk3b, Gucy1a2, Gucy1a3, Gucy1b3, Gucy2c, Gucy2d, Gucy2ep, Gucy2f, Hras, Ilk, Itga1, Itga10, Itga11, Itga2, Itga2b, Itga3, Itga4, Itga5, Itga6, Itga7, Itga8, Itga9, Itgad, Itgae, Itgal, Itgam, Itgav, Itgax, Itgb1, Itgb2, Itgb3, Itgb4, Itgb5, Itgb6, Itgb7, Itgb8, Jam2, Jam3, Jup, Keap1, Kras, Magi2, Magi3, Map2k1, Map2k2, Map2k3, Map2k4, Map2k5, Map2k6, Map2k7, Map3k2, Map3k3, Map3k4, Map3k5, Mapk1, Mapk10, Mapk11, Mapk12, Mapk13, Mapk14, Mapk3, Mapk8, Mapk9, Mllt4, Mphosph6, Mpp6, Mras, Mtmr2, Myo7a, Nanos1, Nanos3, Nos1, Nos2, Nos2p1, Nos2p2, Nos3, Npr1, Npr2, Nprl2, Nras, Ocln, Pdcd6ip, Pls1, Potef, Ppap2b, Prkaca, Prkacb, Prkacg, Prkar1a, Prkar1b, Prkar2a, Prkar2b, Prkg1, Prkg2, Prr3, Pten, Pvrl1, Pvrl2, Pvrl3, Pvrl4, Rab8b, Rac1, Rac2, Rac3, Raf1, Rnase1, Rras, Rras2, Sorbs1, Spta1, Sptan1, Sptb, Sptbn1, Sptbn2, Sptbn4, Sptbn5, Src, Sympk, Tgfb3, Tgfbr3, Tjap1, Tjp1, Tjp2, Tjp3, Tnf, Tnfrsf1a, Tuba3c, Tuba3d, Tuba4a, Tuba8, Tubb, Tubb1, Tubb2a, Tubb3, Tubb4a, Tubb4b, Tubb7p, Tubd1, Tube1, Tubg1, Tubg2, Vcl, Vps72, Was, Wdr1, Ybx3, Zak*

**Germ Cell-Sertoli Cell Junction Dynamics**

*A2m, Acta1, Acta2, Actb, Actg1, Actg2, Actn1, Actn2, Actn3, Actn4, Akt1, Axin1, Bcar1, Bp2, Cdc42, Cdh1, Cdh2, Ctnna1, Ctnna2, Ctnna3, Ctnnb1, Ctnnd1, Diras3, Ensg00000183311, Ensg00000224156, Ensg00000227739, Ensg00000229684, Ensg00000232421, Ensg00000232575, Ensg00000235067, Epn1, Epn2, Epn3, Feom3, Fer, Gsn, Hras, Ilk, Iqgap1, Itgb1, Jup, Keap1, Kras, Lamc3, Lefty2, Limk1, Limk2, Map2k1, Map2k2, Map2k3, Map2k4, Map2k5, Map2k6, Map2k7, Map3k2, Map3k3, Map3k4, Map3k5, Mapk1, Mapk10, Mapk11, Mapk12, Mapk13, Mapk14, Mapk3, Mapk6, Mapk8, Mapk9, Mllt4, Mras, Mtmr2, Myo7a, Nras, Pak1, Pak2, Pak3, Pak4, Pak6, Pak7, Pdpk1, Pik3r1, Pik3r2, Pik3r3, Pik3r4, Pik3r5, Pkn2, Pls1, Potef, Ppap2b, Prr3, Ptk2, Ptk2b, Pvrl2, Pvrl3, Pxn, Rab8b, Rac1, Rac2, Rac3, Rhoa, Rhob, Rhoc, Rhod, Rhog, Rhoh, Rhoj, Rhoq, Rnase1, Rnd2, Rnd3, Rras, Rras2, Sorbs1, Src, Tgfb1, Tgfb2, Tgfb3, Tgfbr1, Tgfbr2, Tjp1, Tnf, Tnfrsf1a, Tuba3c, Tuba3d, Tuba4a, Tuba8, Tubb, Tubb1, Tubb2a, Tubb3, Tubb4a, Tubb4b, Tubb7p, Tubd1, Tube1, Tubg1, Tubg2, Vcl, Was, Zyx*

**Epithelial Tight Junctions**

*Acta1, Acta2, Actb, Actg1, Actg2, Actn1, Actn2, Actn3, Actn4, Actr2, Actr3, Actrt1, Akt1, Akt2, Akt3, Arhgef2, Arl6ip1, Atf1, Atf2, Atf4, Atf5, Atf6, Atf7, Bcar1, Bp2, Cask, Cdc42, Cdk4, Cebpa, Cgn, Cldn1, Cldn10, Cldn11, Cldn12, Cldn14, Cldn15, Cldn16, Cldn17, Cldn18, Cldn19, Cldn2, Cldn20, Cldn23, Cldn3, Cldn4, Cldn5, Cldn6, Cldn7, Cldn8, Cldn9, Clec18c, Crb1, Crb3, Csn3, Cstf1, Cstf2, Cstf2t, Cstf3, Ctnna1, Ctnna2, Ctnna3, Ctnnb1, Cttn, Dab2ip, Ensg00000183311, Ensg00000224156, Ensg00000227739, Ensg00000229684, Ensg00000232421, Ensg00000232575, Ensg00000235067, Epb41, Exoc1, Exoc2, Exoc3, Exoc4, Exoc5, Exoc6, Exoc7, Exoc8, F11r, Feom3, Fos, Fosb, Fosl1, Fosl2, Gna12, Gosr1, Gosr2, Gsk3a, Gsk3b, Hras, Hsf1, Ilk, Inadl, Itga1, Itga10, Itga11, Itga2, Itga2b, Itga3, Itga4, Itga5, Itga6, Itga7, Itga8, Itga9, Itgad, Itgae, Itgal, Itgam, Itgav, Itgax, Itgb1, Itgb2, Itgb3, Itgb4, Itgb5, Itgb6, Itgb7, Itgb8, Jam2, Jam3, Jun, Junb, Jund, Kras, Lefty2, Llgl1, Llgl2, Lnx1, Magi1, Magi2, Magi3, Mapk10, Mapk8, Mapk9, Mark2, Mfsd7, Mllt4, Mpdz, Mphosph6, Mpp5, Mpp6, Mras, Mupp, Myh1, Myh10, Myh11, Myh13, Myh14, Myh15, Myh2, Myh3, Myh4, Myh6, Myh7, Myh7b, Myh8, Myh9, Myl1, Myl12a, Myl12b, Myl2, Myl3, Myl4, Myl5, Myl6, Myl7, Myl9, Mylpf, Myo10, Myo15a, Myo18b, Myo1a, Myo1b, Myo1c, Myo1d, Myo1e, Myo1f, Myo1g, Myo3a, Myo3b, Myo5a, Myo5c, Myo6, Myo7a, Myo9a, Myo9b, Nras, Ocln, Pard3, Pard6a, Pdcd6ip, Potef, Ppap2b, Ppp2ca, Ppp2cb, Ppp2r1a, Ppp2r1b, Ppp2r2a, Ppp2r2b, Ppp2r2c, Ppp2r3a, Ppp2r3b, Ppp2r4, Ppp2r5a, Ppp2r5b, Ppp2r5c, Ppp2r5e, Prkaca, Prkacb, Prkacg, Prkar1a, Prkar1b, Prkar2a, Prkar2b, Prkci, Prkcz, Pten, Rab10, Rab11a, Rab11b, Rab13, Rab14, Rab15, Rab17, Rab18, Rab1a, Rab1b, Rab20, Rab21, Rab22a, Rab23, Rab24, Rab25, Rab26, Rab27a, Rab27b, Rab28, Rab2a, Rab2b, Rab30, Rab31, Rab32, Rab33a, Rab33b, Rab34, Rab3a, Rab3b, Rab3c, Rab3d, Rab43, Rab4a, Rab4b, Rab5a, Rab5b, Rab5c, Rab6a, Rab6b, Rab6c, Rab7a, Rab7b, Rab8a, Rab8b, Rab9a, Rab9b, Rac1, Rhoa, Rnase1, Rnaseh2a, Rras, Rras2, Safb, Sec22b, Smurf1, Snap23, Snap25, Spta1, Sptan1, Sptb, Sptbn1, Sptbn2, Sptbn4, Sptbn5, Stx1a, Stx1b, Stx2, Stx3, Stx4, Stx5, Sympk, Tgfb1, Tgfb2, Tgfb3, Tgfbr1, Tgfbr2, Tgfbr3, Tiam1, Tjap1, Tjp1, Tjp2, Tjp3, Tnf, Tnfrsf1a, Tuba3c, Tuba3d, Tuba4a, Tuba8, Tubb, Tubb1, Tubb2a, Tubb3, Tubb4a, Tubb4b, Tubb7p, Tubd1, Tube1, Tubg1, Tubg2, Vamp1, Vamp2, Vamp3, Vapa, Vasp, Vcl, Wdr1, Ybx3, Zak*

**Regulation of Microtubule Cytoskeleton**

*Abl1, Akt1, Apc, Aurkb, Camk4, Cdc42, Cdk1, Cfl2, Clasp1, Clip1, Dpysl2, Dvl1, Ephb2, Gnaq, Gsk3b, Kif2c, Limk1, Map1b, Mapkapk2, Mapre1, Mapt, Mark1, Mark2, Pak1, Pard6a, Phldb2, Pik3ca, Prkaca, Prkca, Pten, Ptpra, Rac1, Rho, Rock1, Spred1, Src, Stat3, Stmn1, Taok1, Tesk2, Tiam1, Tppp, Trio, Wnt3a*

**Regulation of actin cytoskeleton SuperPath**

*Abi2, Actb, Actg1, Actn1, Actn2, Actn3, Actn4, Apc, Apc2, Araf, Arhgap35, Arhgef1, Arhgef12, Arhgef4, Arhgef6, Arhgef7, Arpc1a, Arpc1b, Arpc2, Arpc3, Arpc4, Arpc5, Arpc5l, Baiap2, Bcar1, Bdkrb1, Bdkrb2, Braf, Brk1, Cd14, Cdc42, Cfl1, Cfl2, Chrm1, Chrm2, Chrm3, Chrm4, Chrm5, Crk, Crkl, Csk, Cyfip1, Cyfip2, Diaph1, Diaph2, Diaph3, Dock1, Egf, Egfr, Enah, Ezr, F2, F2r, Fgd1, Fgd3, Fgf1, Fgf10, Fgf11, Fgf12, Fgf13, Fgf14, Fgf16, Fgf17, Fgf18, Fgf19, Fgf2, Fgf20, Fgf21, Fgf22, Fgf23, Fgf3, Fgf4, Fgf5, Fgf6, Fgf7, Fgf8, Fgf9, Fgfr1, Fgfr2, Fgfr3, Fgfr4, Fn1, Git1, Gna12, Gna13, Gng12, Gsn, Hras, Ins, Insrr, Iqgap1, Iqgap2, Iqgap3, Itga1, Itga10, Itga11, Itga2, Itga2b, Itga3, Itga4, Itga5, Itga6, Itga7, Itga8, Itga9, Itgad, Itgae, Itgal, Itgam, Itgav, Itgax, Itgb1, Itgb2, Itgb3, Itgb4, Itgb5, Itgb6, Itgb7, Itgb8, Kras, Limk1, Limk2, Loc648044, Map2k1, Map2k2, Mapk1, Mapk3, Mapk4, Mapk6, Mos, Mras, Msn, Myh10, Myh14, Myh9, Myl1, Myl10, Myl12a, Myl12b, Myl2, Myl3, Myl5, Myl7, Myl9, Mylk, Mylk2, Mylk3, Mylk4, Mylpf, Nckap1, Nckap1l, Nras, Pak1, Pak2, Pak3, Pak4, Pak6, Pak7, Pdgfa, Pdgfb, Pdgfc, Pdgfd, Pdgfra, Pdgfrb, Pfn1, Pfn2, Pfn3, Pfn4, Pik3c2a, Pik3c2b, Pik3c2g, Pik3c3, Pik3ca, Pik3cb, Pik3cd, Pik3cg, Pik3r1, Pik3r2, Pik3r3, Pik3r4, Pik3r5, Pikfyve, Pip4k2a, Pip4k2b, Pip4k2c, Pip5k1a, Pip5k1b, Pip5k1c, Pip5kl1, Ppp1ca, Ppp1cb, Ppp1cc, Ppp1r12a, Ppp1r12b, Ppp1r12c, Ptk2, Pxn, Rac1, Rac1p2, Rac1p4, Rac2, Rac3, Raf1, Rassf7, Rdx, Rhoa, Rock1, Rock2, Rras, Rras2, Scin, Slc9a1, Sos1, Sos2, Src, Ssh1, Ssh2, Ssh3, Tiam1, Tmsb4x, Tmsb4y, Vav1, Vav2, Vav3, Vcl, Vil1, Was, Wasf1, Wasf2, Wasl*

**Myosin Family**

*Myo15, Myo16, Myo18a, Myo18b, Myo19, Myo1a, Myo1b, Myo1c, Myo1d, Myo1e, Myo1f, Myo1g, Myo5a, Myo5b, Myo6, Myo7a, Myo7b, Myo9a, Myo9b, Myoc, Myocd, Myof, Myom2, Myom3, Myoz1, Myoz2*

**Cell-extracellular matrix interactions**

*Arhgef6, Pxn, Ilk, Itgb1, Actg1, Actb, Actn1, Vasp, Lims2, Lims1, Parvb, Parva, Flna, Flnc, Fermt2, Fblim1, Rsu1, Tesk1*

**Cell adhesion molecules**

*Alcam, Cadm1, Cadm3, Cd2, Cd22, Cd226, Cd274, Cd276, Cd28, Cd34, Cd4, Cd40, Cd40lg, Cd58, Cd6, Cd80, Cd86, Cd8a, Cd8b, Cd99, Cdh1, Cdh15, Cdh2, Cdh3, Cdh4, Cdh5, Cldn1, Cldn10, Cldn11, Cldn14, Cldn15, Cldn16, Cldn17, Cldn18, Cldn19, Cldn2, Cldn20, Cldn22, Cldn23, Cldn24, Cldn25, Cldn3, Cldn4, Cldn5, Cldn6, Cldn7, Cldn8, Cldn9, Cntn1, Cntn2, Cntnap1, Cntnap2, Ctla4, Esam, F11r, Glg1, Hla-a, Hla-b, Hla-c, Hla-dma, Hla-dmb, Hla-doa, Hla-dob, Hla-dpa1, Hla-dpb1, Hla-dqa1, Hla-dqa2, Hla-dqb1, Hla-dra, Hla-drb1, Hla-drb3, Hla-drb4, Hla-drb5, Hla-e, Hla-f, Hla-g, Icam1, Icam2, Icam3, Icos, Icoslg, Itga4, Itga6, Itga8, Itga9, Itgal, Itgam, Itgav, Itgb1, Itgb2, Itgb7, Itgb8, Jam2, Jam3, L1cam, Loc100509457, Loc101060835, Loc101929889, Lrrc4, Lrrc4b, Lrrc4c, Madcam1, Mag, Mpz, Mpzl1, Ncam1, Ncam2, Negr1, Neo1, Nfasc, Nlgn1, Nlgn2, Nlgn3, Nlgn4x, Nrcam, Nrxn1, Nrxn2, Nrxn3, Ntng1, Ntng2, Ocln, Pdcd1, Pdcd1lg2, Pecam1, Ptprc, Ptprf, Ptprm, Pvr, Pvrl1, Pvrl2, Pvrl3, Sdc1, Sdc2, Sdc3, Sdc4, Sele, Sell, Selp, Selplg, Siglec1, Spn, Tigit, Vcam1, Vcan, Vtcn1*
